# Autoimmune Hepatitis: From Evolution to Current Status—A Pathologist’s Perspective

**DOI:** 10.3390/diagnostics14020210

**Published:** 2024-01-18

**Authors:** Puja Sakhuja, Surbhi Goyal

**Affiliations:** Department of Pathology, Govind Ballabh Pant Institute of Postgraduate Medical Education and Research, New Delhi 110002, India; dr.surbhi4you@gmail.com

**Keywords:** autoimmune hepatitis, International Autoimmune Hepatitis Group, simplified diagnostic criteria, histology, plasma cell clusters, lobular hepatitis, portal hepatitis

## Abstract

Autoimmune hepatitis (AIH) is a chronic, relapsing and remitting, immune-mediated liver disease that progresses to cirrhosis if left untreated. A significant number of patients may present with acute hepatitis or acute liver failure, which are often misdiagnosed as toxic liver injury. AIH shows a preponderance in young women but may be seen in children and the elderly. Diagnosis requires the integration of clinical, biochemical, and serologic parameters, along with supportive liver histology and exclusion of other causes of liver disease. Liver biopsy is a prerequisite for diagnosis of AIH, to assess severity and stage of disease, exclude other entities, and recognize any concurrent morbidities. No single biomarker or histologic feature is pathognomonic for AIH. The diagnostic and histologic criteria have undergone several modifications since the original scoring system was proposed by the International Autoimmune Hepatitis Group (IAIHG) in 1993. Recently, the IAIHG has proposed consensus recommendations for histologic criteria, relevant for both acute and chronic AIH. This review article will describe the evolving diagnostic criteria for AIH, with their limitations and utility, and with an emphasis on the role of liver histology in the diagnosis and management of AIH.

## 1. Introduction

Autoimmune hepatitis (AIH) is a chronic, relapsing and remitting, immune-mediated inflammatory disorder of the liver. AIH presents in young females more frequently, although it can manifest in any age group, ranging from infancy to the eighth decade. Presentation can be extremely heterogenous, from being asymptomatic to acute on chronic hepatitis, chronic hepatitis, and rarely acute fulminant hepatitis. Around one third of patients present with cirrhosis due to undetectable subclinical disease. The diagnosis of AIH is essentially a multidisciplinary diagnosis involving clinical, biochemical, serologic, and histologic criteria. AIH is associated with tissue-directed autoantibodies and hypergammaglobulinemia. Timely diagnosis and appropriate therapy can be lifesaving, as the disorder usually responds well to immunosuppressive therapy. As there is no single pathognomonic or diagnostic feature, the criteria for diagnosis of AIH have seen many revisions over the last few decades. This article aims to describe the evolution in the diagnostic criteria of AIH, with special emphasis on the histologic criteria.

### Epidemiology

AIH has a global annual incidence of 1.37 per 100,000 and prevalence of 17.44 per 100,000, with a significant female preponderance (prevalence of 12.77 for females and 2.91 for males) [1]. The pooled prevalence rate in Asian (12.99/100,000) is lower than that in European (19.44/100,000) and American populations (22.80/100,000) [2].

Epidemiological data on AIH in India is scant. In 2015, Amarapurkar et al. reported a prevalence of 1.3% amongst all liver disease patients and 8.74% in chronic liver disease [3].

## 2. Diagnosis

Diagnosis of autoimmune hepatitis relies on clinical history, biochemical findings in the presence of raised IgG and positive serum autoantibodies, and favorable histology. Documenting the absence of other chronic liver disease such as viral hepatitis, alcoholic or non-alcoholic steatohepatitis, and Wilson’s disease is required. Occasionally, only close observation of the response to steroid therapy or a relapse of disease upon dose reduction or discontinuation of therapy allows making the final diagnosis.

### 2.1. Hypergammaglobulinema

Selective elevation of IgG with normal levels of IgA and IgM is a characteristic feature of AIH and is seen in around 90% of chronic AIH patients. A few patients have IgG levels in the upper range of normal (relative increase), which fall upon immunosuppressive therapy.

### 2.2. Serum Autoantibodies

Presence of autoantibodies remains the hallmark of AIH diagnosis in all scoring systems. Based on serology, AIH can be classified into (1) type I AIH with ANA and/or ASMA and (2) type II AIH with anti-LKM-1 and/or anti-liver cytosol type-1 antibodies [4] (Table 1).

### 2.3. Absence of Viral Hepatitis

It is nearly impossible to make a histologic distinction between viral hepatitis and AIH. Raised levels of IgG and serum autoantibodies are quite frequently observed in viral hepatitis. Thus, on their own, they are insufficient for establishing a diagnosis of AIH. This is especially relevant in view of the high prevalence (10–40%) of hepatitis B and C in Southeast Asia and Africa.

## 3. Early Descriptions

One of the earliest publications to describe what we now know as AIH, reported it as a persistent, relapsing jaundice with a preponderance in young females, less than 30 years of age, and raised serum globulins [5]. It was recognized as an ‘anomaly of immune tolerance’, with viral hepatitis as a trigger. The resemblance to lupoid hepatitis and progression to cirrhosis was described [6].

Histologically numerous lymphoid cells, dense fibrosis, and a ‘remarkable degree of plasma cell infiltration of the liver’ was described [7]. The immune nature of the disease was noted by several subsequent publications, and the term lupoid hepatitis and autoimmune hepatitis were used to describe the condition [8,9].

## 4. Histology of Autoimmune Hepatitis

Histologic evidence of hepatitis remains the cornerstone of AIH diagnosis, along with clinical and serologic parameters. Recent guidelines recommend liver biopsy at presentation to establish the diagnosis of AIH, to exclude other differentials, highlight any concurrent comorbidities, grade the necroinflammatory activity for taking treatment decisions, and evaluate the extent of fibrosis (staging). It is noteworthy that liver histology may be of prognostic significance in terms of progression to cirrhosis or malignancy. Autoimmune hepatitis is histologically a form of chronic hepatitis, thus having portal inflammation, interface activity, and lobular inflammation. However, a few features have been described as more commonly seen in AIH, which have been used in the development of scoring systems (described later in this article).

### 4.1. Portal Inflammation

**Figure 1:**
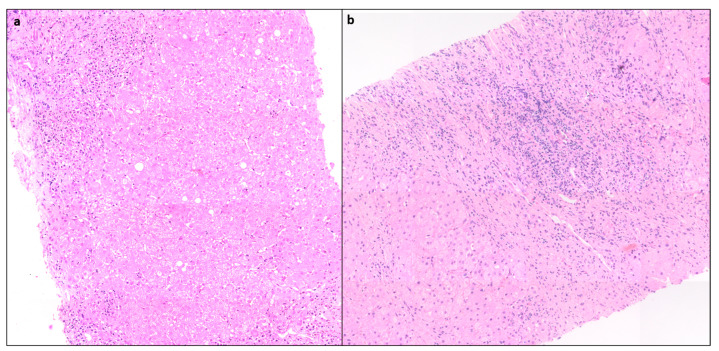
(**a**) Case of AIH showing moderate interface activity and portal inflammation, (mHAI:Interface activity (A) = 2, Confluent necrosis (B) = 0, lobular inflammation (C) = 0, D (portal inflammation) = 2, H&Ex 80). (**b**) Another case showing moderate portal inflammation, interface activity, and spotty necrosis (mHAI: A = 3, B = 0, C = 2 and D = 3, H&E × 100).

Portal inflammation (PI) is usually mononuclear consisting of lymphocytes, plasma cells, and some histiocytes. Few neutrophils and eosinophils can be seen. PI can range from mild to severe but is often moderate to severe grade [10] (Figure 1).

### 4.2. Interface Hepatitis

Interface hepatitis with hepatocyte necrosis of the periportal parenchyma is a classic histological finding seen in more than 85% of cases and can range from mild to severe [11] (Figure 1). In AIH with severe activity, the PI and interface activity can be associated with bridging necrosis. It is often more severe in AIH than in chronic hepatitis of other etiologies.

### 4.3. Lobular Inflammation (LI) or Hepatocyte Necrosis

**Figure 2:**
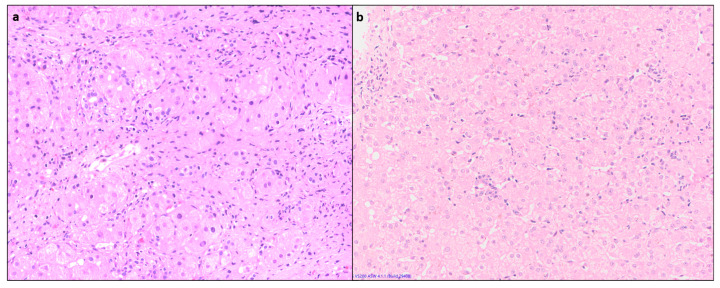
(**a**) Liver biopsy showing foci of spotty necrosis and lobular inflammation: (**a**) mHAI- C = 3, (**b**) mHAI- C = 2 (H&E × 200).

**Figure 3:**
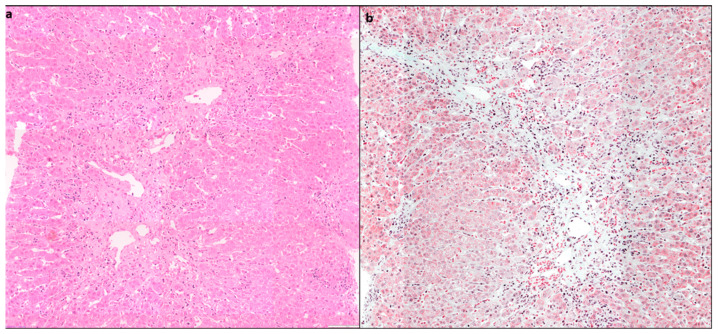
A case of acute AIH showing centrilobular necrosis and perivenulitis, confirmed by absence of fibrosis on Masson’s trichrome: (**a**) H&E, (**b**) (MT × 100).

Lobular inflammation of variable severity is usually present, ranging from scattered necroinflammatory foci (spotty necrosis) to confluent and/or bridging necrosis (Figure 2). This may be associated with lobular disarray, ballooning degeneration, and hepatocyte regeneration. Large areas of confluent necrosis can be seen, especially in acute fulminant presentation or during disease flares [10]. Centrilobular inflammation and necrosis may be prominent in about one third of cases [12] (Figure 3). Rarely, this may be the predominant form of injury in acute AIH.

### 4.4. Plasma Cell Predominance

**Figure 4:**
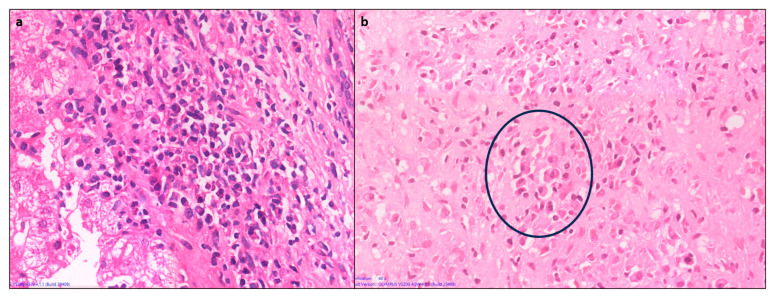
(**a**) Liver biopsy showing dense portal lymphoplasmacytic inflammation comprising predominantly plasma cells in sheets and clusters (>5 cells), along with lymphocytes (H&E × 400), (**b**) another case of AIH with areas of centrilobular confluent necrosis, showing plasma cell clusters (highlighted in circle) along with RBC extravasation and hepatocyte loss (H&E × 200).

Plasma cells are prominent in about two thirds of cases of AIH. Plasma cell clusters, defined as a collection of five or more plasma cells, have been noted in AIH [10,13] (Figure 4).

### 4.5. Hepatocyte Rosettes

**Figure 5:**
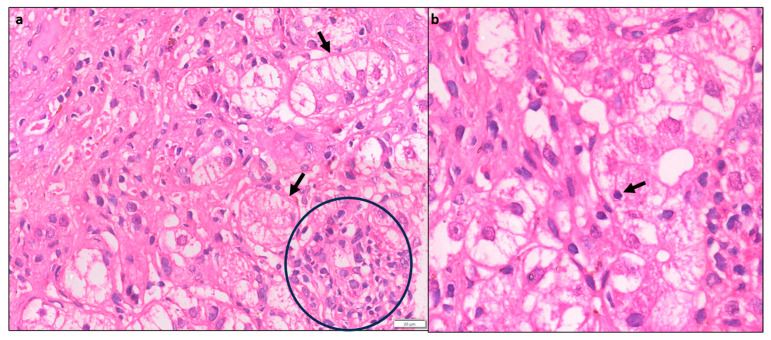
(**a**) A case of AIH showing prominent resetting of hepatocytes (black arrows) in the periportal area, along with a focus of lymphocytic lobular inflammation (circle) (H&E × 200). (**b**) Higher magnification showing an intact lymphocyte within the hepatocyte (emperipolesis) (H&E × 400).

Rosettes are a regenerative feature where the hepatocytes are arranged around a small, sometimes not visible lumen (Figure 5). They can be seen following hepatocellular injury of any cause and may indicate severe injury. Thus, rosettes and emperipolesis may be seen more frequently in AIH (49%) compared to chronic viral hepatitis [13].

### 4.6. Emperipolesis

Emperipolesis is a lymphocyte or rarely a plasma cell within the cytoplasm of a hepatocyte (Figure 5). This finding has been reported in 65–78% of AIH biopsies [13]. The lymphocytes are usually CD8+ T lymphocytes [14]. Emperipolesis may induce hepatocyte apoptosis and thus may be associated with higher serum transaminase levels and more severe disease activity. Emperipolesis has also been described in drug-induced liver injury, primary biliary cirrhosis, and chronic viral hepatitis; therefore, it is not pathognomonic for AIH [10,13].

### 4.7. Giant Cell Hepatitis

Around 40% cases of adult post infantile giant cell hepatitis may be associated with autoimmune hepatitis, where there is prominent giant cell transformation of hepatocytes [15]. This is a progressive disease, with survival rate of 50% without transplantation [16].

### 4.8. Cirrhosis

Around one-third of AIH patients have cirrhosis at the time of presentation, which is usually of the macronodular type [17]. AIH-related cirrhosis does not have any pathognomonic histological features. Hepatocellular carcinoma is rarely documented in the context of cirrhosis only, with an incidence of 0.3–1% per year [18].

## 5. Development of Scoring Systems

Diagnostic scores help clinicians in making a primary clinical diagnosis, by weighing the different laboratory, serological, and histological results. In addition, diagnostic scores are important in stratifying patients by defined unified criteria and help in making scientific studies comparable.

### 5.1. Original Scoring System of the International Autoimmune Hepatitis Group (IAIHG), 1993

This led to the development of the diagnostic scoring system of the International Autoimmune Hepatitis Group (IAIHG), created by an international panel in 1993 [19]. In addition to liver histology (Box1), they defined minimum required parameters related to serum biochemistry, serum immunoglobulins, serum autoantibodies, viral markers, and absence of other etiological factors, in order to arrive at a ‘definite’ or ‘probable’ diagnosis of AIH. A scoring system for diagnosis of autoimmune hepatitis was defined, with positive and negative weighted scores assigned to various parameters. Features of liver histology were assigned specific scores (Table 2), as additional features and not minimum required parameters. The system was designed to be applied to treatment-naïve and post-treatment liver biopsies.

Liver histology was described as a chronic active hepatitis. In addition, they acknowledged that intense activity, presence of numerous plasma cells, and liver cell rosettes, although suggestive, were not pathognomonic of AIH [19].

### 5.2. Revised IAIHG Modified Scoring System, 1999

Over time, it became evident that using the 1993 criteria, patients with other autoimmune biliary conditions such as primary sclerosing cholangitis (PSC) and primary biliary cirrhosis (PBC) could be ascribed a false-positive diagnosis of AIH. In 1999, the 40 member IAIHG revised the criteria, in order to obtain greater sensitivity for the diagnosis of AIH and to be able to exclude non-AIH disorders, especially biliary disease such as PBC and PSC [20,21]. The principal changes in the assigned scores were in the ALP:AST (or ALT) ratio, drug history, liver histology, and response to therapy. It was also emphasized that the system was intended mainly for research purposes but may be useful in the diagnosis of difficult cases.

Both the 1993 and 1999 revised criteria clearly implied: (1) liver histology is an important component in the diagnosis of AIH [22], (2) while features typical of AIH such as female gender, raised AST, ALT, and presence of autoantibodies help in making a diagnosis, this scoring system helps rule out other differentials by assigning negative scores to features such as positive viral serology, use of drugs or alcohol, etc. It is important to remember that autoantibodies can be positive in several other chronic liver diseases, including hepatitis C and NASH [23]. One of the challenges that remains with these scoring systems, is the diagnosis of AIH when an associated comorbidity is present (such as NASH) or in the diagnosis of AIH overlap syndrome [24]. In such cases, liver histology plays an important role. The IAIHG reiterated the importance of a liver biopsy, preferably seen by a hepatopathologist, to arrive at a definite diagnosis of AIH [10].

However, these criteria were complex, included a variety of parameters of questionable value, and their practical utility for daily clinical use remained challenging, including 13 categories, some of them impractical in children.

### 5.3. Simplified Scoring System (SSS) for AIH, 2000

To overcome these difficulties in the revised scoring system (1999), IAIHG decided to devise a simplified scoring system for wider applicability in routine clinical practice based on the data of patients with well-established diagnoses. They studied various clinical, biochemical, and serologic data, based on the opinion of the experts of the IAIHG. A training set consisting of the data from 250 patients with proven AIH and 193 controls was included [25]. The validation set included 109 patients with AIH and 284 controls. The controls included PBC, PSC, NASH, viral hepatitis, Wilson’s disease, hemochromatosis, and drug/toxin-induced liver injury. From these data, they developed a simple scoring system that included autoantibodies (ANA, ASMA, LKM, and/or SLA), total IgG level, absence of viral hepatitis, and liver histology (Table 3). While in the 1999 revised criteria for autoimmune hepatitis, cholestatic features on histology excluded the diagnosis of autoimmune hepatitis, the SSS deliberately included these patients, to ensure that they receive immunosuppressive treatment if the clinical suspicion of AIH is high. The simplified diagnostic criteria scoring system reported a sensitivity and specificity of 88% and 97%, respectively, for a score of 6 points, defined as ‘probable AIH’; and 81% and 99%, respectively, for a score of 7 or more points, defined as ‘definite AIH’ [25]. Subsequent studies revealed a sensitivity for a score of >6 from 65% to 95%, and for a score of 7 or more from as low as 15% up to 87%; with a positive predictive value of 83–100% and a negative predictive value of 74% to 97% [26].

Histology was included as essential for the diagnosis of AIH, even though it may not show lesions typical for AIH. Demonstration of hepatitis on histology was considered a prerequisite. Professors Dienes and Lohse defined the liver histology under three categories: ‘typical’, ‘compatible’, and ‘atypical’ (Table 4). The importance of liver histology lay not just in establishing the diagnosis and the severity of AIH, but also to exclude other disease entities like biliary pathology and identify comorbidities like NASH, which is being increasingly recognized nowadays. The main advantage of the SSS was its simplicity for use in everyday practice.

Soon after these criteria were published, Yeoman et al. evaluated the diagnostic accuracy, utility, and comparative evaluation of the simplified score (SSS) in 549 patients with chronic liver disease, of which 221 were AIH and 26 were variant syndromes. They found a concordance rate of 90% with 1999 criteria for probable and 61% for definite AIH. The simplified criteria had high specificity (98% for score >/= 6 and 100% for scores >/= 7), but exhibited lower sensitivity for scores of >/= 7 (70%). The authors felt that the low sensitivity of SSS may have been due to the omittance of discriminating information such as response to corticosteroids. Furthermore, they found a lower frequency (24%) of overall diagnosis of AIH (probable or definite AIH) among the 70 patients with fulminant liver failure for SSS and 40% for 1999 criteria [26]. In addition, as the diagnosis of ‘probable’ AIH is based on clinical and laboratory evaluations and not on treatment response, these patients should not be denied treatment or inclusion in clinical studies if the clinician’s index of suspicion is strong [27].

De Boer et al. found emperipolesis and rosette formation to be superior histological predictors of AIH than interface hepatitis and plasma cells. In addition, they found moderate to severe lymphocytic cholangitis in 28% of AIH patients [13]. However, others have argued that cases of autoimmune hepatitis, in the absence of rosettes and emperipolesis, would only receive a histologic score of 1 point, which might result in lower sensitivity [28]. In addition, these features showed marked interobserver variability and inconsistency in histologic interpretation.

The drawbacks of the simplified criteria, which were gradually recognized, included lack of sensitivity for diagnosis of acute AIH or AIH with comorbidities [10,29,30].

In a recent study in patients with acute onset AIH, the diagnostic performance of the two AIH scoring systems was assessed. The researchers found that the revised version of the original criteria (1999) achieved a diagnosis of AIH in about 30% of patients as compared to the simplified score [31]. This was predominantly due to the normal serum IgG levels and lower frequency of ANA and SMA positivity seen in these patients. Furthermore, Joshita et al. reported that SSS is unreliable for diagnosis of acute AIH. More than half of AIH patients with acute presentation had a normal IgG level, and the prevalence of seropositivity for ANA and SMA was reported to be 73% and 28%, respectively [32]. Though more investigative and time consuming, the revised version of the original criteria (1999) has higher sensitivity in diagnosing atypical AIH patients.

Furthermore, emperipolesis and resetting are features that indicate hepatic injury and regeneration in the background of hepatitis and, therefore, are not specific to AIH. When comparing AIH with grade matched HCV as controls, Gurung et al. did not find any significance for these two histologic features in diagnosis of AIH [33].

### 5.4. Histologic Scoring of Autoimmune Hepatitis by Balitzer et al. [28]

Addressing the drawbacks of the SSS, Balitzer et al. compared the histologic criteria of the SSS to a revised set of histologic criteria in 88 cases of autoimmune hepatitis, 20 cases of primary biliary cholangitis, and 13 cases of non-autoimmune acute hepatitis. They used histologic scores as given in Table 5. Their study showed an increase in the number of cases having a score of >6 using their proposed histologic criteria from 69% to 86% in cases of AIH. The histologic criteria proposed highlight the degree of hepatitic activity (interface and/or lobular) and prominence of plasma cells. Furthermore, a histologic score of 1 required the absence of cholestatic features on copper (positive copper or copper associated protein) and CK7 stains (CK7 positive periportal hepatocytes), thus differentiating from chronic biliary disease [28].

Using the histologic criteria of the SSS, the diagnosis of acute hepatitis had poor sensitivity. Using the revised criteria, the authors demonstrated an improved sensitivity for probable/definite auto- immune hepatitis from 62% to 90% [28]. This would facilitate timely immunosuppressive therapy, which could be lifesaving or prevent rapid progression of fibrosis.

### 5.5. Modified Scoring Criteria Proposed by Gurung et al. [33]

**Figure 6:**
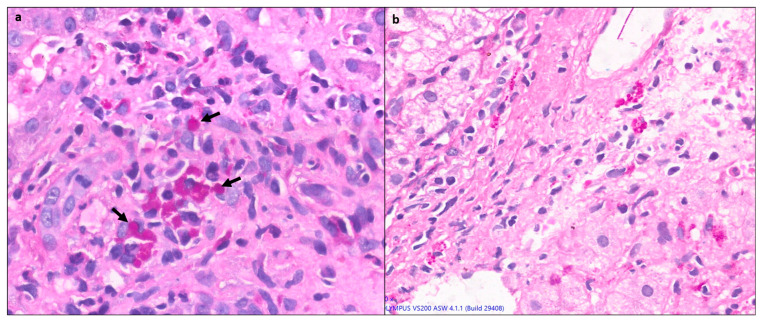
(**a**) A case of AIH showing characteristic pink hyaline globules (black arrows) within the Kupffer cells in the portal area (PAS with diastase ×400). (**b**) For comparison, Kupffer cells showing PAS-positive diastase-resistant ceroid pigment in chronic hepatitis (×400).

Gurung et al. conducted a critical appraisal on the histologic features of AIH and found plasma-lymphocytic inflammation, defined as plasma cell predominance, and plasma cell clusters (>/=5 plasma cells) were the most reliable distinguishing features in AIH. Another histologic feature evaluated is Kupffer cell hyaline globules (KcHG) [33,34]. KcHG are defined as round, glassy, well defined/sharply circumscribed intracytoplasmic structures, best seen on Periodic acid Schiff with diastase (PASD) stain, and can be differentiated from ill-defined PAS-D granules in Kupffer cells [34] (Figure 6). Using these criteria (Table 6), they found a high positive predictive value (70–88%) [34].

### 5.6. Consensus Recommendations for Histological Criteria of AIH from IAIHG [35]

The proposed histologic criteria for making a diagnosis of AIH over the years have largely been based on old studies and have neither been prospectively validated, nor agreed upon by international consensus. These criteria were focused predominantly on the portal-based inflammation seen in chronic AIH, and cases of acute AIH with lobular-based inflammation were often misclassified as drug-induced or toxic acute liver injury [35].

Considering these concerns about the histologic criteria of the SSS and the subsequent studies and recommendations, the European Reference Network on Hepatological Diseases and the European Society of Pathology hosted a workshop on AIH histology in Brussels, Belgium, in January 2020 [25].

The participants were 17 liver pathologists and two hepatologists with expertise in AIH. They proposed in their consensus statement criteria for the histological diagnosis of AIH in the native liver, which would apply to patients with an acute as well as a chronic presentation [35] (Table 7).

Guidelines in this recommendation include the standards for a liver biopsy, which should be performed with a 18/16 G needle, of 1.5 cm length, with at least 6–8, preferably 10, portal tracts. A connective tissue stain is a must to identify the extent and distribution of fibrosis and helps differentiate from areas of necrosis/collapse. Trichrome, orcein, and Sirius red are the stains that can be used. They conducted a Delphi round and agreed upon the following statements [35]:

- AIH has no pathognomonic histological features;
- It is desirable to have the knowledge of the duration of the liver disease before assessing a liver biopsy for AIH;
- Emperipolesis and Rosettes should be discarded as diagnostic features for AIH, owing to limited specificity;
- Evaluating a biopsy for AIH involves assessment of the dominant pattern of inflammation; that is, portal (chronic) hepatitis or lobular (acute) hepatitis;
- The histological spectrum of AIH, especially acute presentation, now recognizes and includes centrilobular injury with prominent hepatocellular necrosis;
- Lymphoplasmacytic inflammatory infiltrate refers to predominance of plasma cells (including plasma cell clusters), in addition to lymphocytes;
- A plasma cell cluster is defined as more than five plasma cells in one focus;
- A comment on the presence and severity of fibrosis should be included in the pathology report;
- The likelihood of AIH should be defined as unlikely, possible, or likely.

They recommend semi-quantitative assessment of the severity of inflammatory activity of AIH based on Ishak’s modified Histological Activity Index (mHAI) [36]. Grading of necro-inflammatory activity and staging of fibrosis in AIH can be performed using the systems developed for chronic viral hepatitis. Simple four-tier grading systems like the Batts and Ludwig [37], Scheuer [38], and Metavir [39] can be used too, but they preferred Ishak’s mHAI, as it allows more detailed assessment of centrilobular necroinflammatory changes and is more appropriate for grading acute lobular damage [35]. The main highlight of this consensus scoring was the inclusion of acute AIH cases as centrilobular injury, with hepatocyte necrosis being recognized as a part of the histologic spectrum of AIH, which till then had been conventionally associated with DILI [35].

The SSS required the exclusion of viral hepatitis; however, the consensus recommendation additionally suggested the exclusion of viral hepatitis through PCR in the setting of acute hepatitis and drug history of the last 6 months. History of previous transaminitis supports a chronic relapsing course of AIH [35].

## 6. Autoantibody Negative AIH

‘Autoantibody-negative’ AIH occurs in 10–20% of AIH cases and is defined by the absence of ANA, ASMA, anti-LKM, and AMA antibodies at the time of presentation. It clinically resembles type 1 AIH and has good response rate to steroids [40]. Liver histology is mandatory for diagnosis in such cases.

## 7. Anti Mitochondrial Antibody-Positive AIH

O’Brien et al. studied 15 patients with AIH who had classical features of AIH on biopsy but were found to be anti-mitochondrial antibody-M2 (AMA)-positive. None of these patients had histological evidence of PBC nor did any of them develop PBC on follow-up [41]. It is controversial whether these patients represent a subtype of AIH or whether treatment with corticosteroids can prevent the subsequent development of PBC or an ‘overlap’ syndrome.

## 8. Overlap Syndrome

The majority of cases of so called ‘overlap syndromes’ involve AIH with PBC or PSC. Histologically focal lymphocytic cholangitis and bile duct injury can be seen in 12–20% of AIH cases; therefore, their presence does not preclude the diagnosis of AIH [10,13]. As per the recent consensus statement, if features of PBC, PSC, or NAFLD are present along with typical features of AIH, a liver biopsy can still be classified as possible AIH. However, if a biopsy from a clinically suspected patient of AIH has unusually prominent biliary features, such as bile duct loss or features of chronic cholestasis (e.g., periportal deposits of copper, copper-associated protein, or periportal keratin 7-positive cells with an intermediate hepatobiliary phenotype), possibility of PBC or PSC with AIH-like features may be considered [35].

For diagnosis of PBC the Paris criteria are recommended, which require two out of three criteria for PBC (florid bile duct lesions on histology, ALP ≥ two times the upper limit of normal or GGT ≥ five times ULN, and AMA antibodies on indirect immunofluorescence), as well as two of three criteria for AIH (ALT ≥ five times ULN, IgG ≥ two times ULN, liver biopsy showing moderate to severe interface activity) [42].

Overlap with PSC shows cholangiographic evidence of focal biliary strictures, in addition to the features of AIH.

## 9. NAFLD (Nonalcoholic Fatty Liver Disease)

Positive autoantibody serology, usually comprising low titers of ANA, ASMA, and/or AMA, has been documented in 20–48% of NAFLD patients [43,44]. Furthermore, hypergammaglobulinemia may be seen in some elderly female patients with nonalcoholic steatohepatitis (NASH)-related cirrhosis and when viral markers are negative. Therefore, patients with NASH may be misdiagnosed as probable AIH using the simplified scoring system, underscoring the importance of liver biopsy in such situations [25,44].

## 10. Concurrence of AIH with Other Liver Diseases

Concurrence of AIH with viral hepatitis is a very challenging situation for clinicians as well as pathologists, since the presence of chronic viral hepatitis is an exclusion criterion for AIH in diagnostic scoring systems. This problem is not uncommon in countries where there is a high prevalence of hepatitis virus B (HBV) or hepatitis virus C (HCV) infection. In addition, increased serum IgG and autoantibodies (ASMA up to 66%, ANA up to 41% in chronic hepatitis C) and overlapping histological features of chronic active hepatitis with periportal piecemeal necrosis and lymphoid follicles in chronic HCV can lead to misdiagnosis [10,44,45]. Muratori et al. rightly pointed out the issue of the LKM1-positivity seen in 38 patients amongst 143 hepatitis C patients. Whether LKM1 antibody titers should only be used in SSS in the absence of HCV infection, remains a topic of debate and requires further study [46].

**Figure 7:**
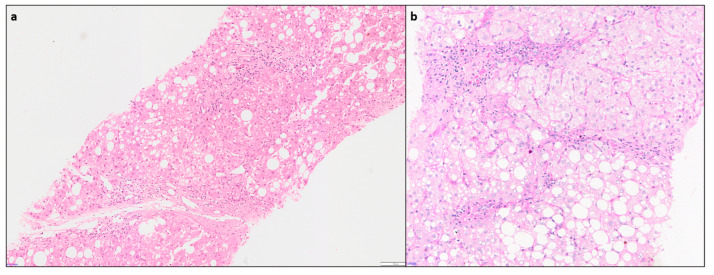
(**a**) Liver biopsy from a clinically suspected case of AIH showing features of concurrent steatohepatitis (H&E × 100), (**b**) higher magnification showing moderate portal hepatitis along with foci of steatohepatitis, which warranted a downgrade of AIH consensus recommendation score from likely to possible AIH (PAS × 150).

Hepatic comorbidities such as NAFLD and NASH are increasingly seen, in 15–30% of AIH cases [44,47]. As missing a diagnosis of AIH, with its therapeutic and prognostic consequences, can be detrimental to the patient, it is important that clinicians are notified of these comorbidities [35]. Diagnosis of AIH with concurrent steatohepatitis (SH) of alcoholic or non-alcoholic etiology can be challenging. Hepatocyte ballooning with rarefaction, Mallory–Denk body and centrilobular ‘chicken- wire’ sinusoidal fibrosis are characteristic features of SH and usually not seen in AIH (Figure 7). Presence of these findings may guide diagnosing concurrent SH in an AIH patient having otherwise typical histological and serological features [10].

## 11. Pediatric Autoimmune Hepatitis

The pediatric age group may have certain limitations in diagnosis, as over half of children have no autoantibodies and do not show hypergammaglobulinemia, which can lead to false-negative results [48,49,50]. Thus, the simplified scoring system has a much lower sensitivity in this age group [49]. Machancoses et al. [50] proposed a simplified diagnostic scoring system after studying 100 cases of pediatric autoimmune hepatitis, which included five criteria: autoantibodies (0–2 points), hypergammaglobulinemia, exclusion of viral hepatitis, exclusion of Wilson’s disease (1 point each), and interface hepatitis or multilobular collapse on liver histology (3 points). In addition, a normal cholangiogram is mandatory. They found a sensitivity and specificity of 95.8% and 100% at a score of ≥6. This new proposal was helpful in diagnosing seronegative AIH when the other criteria were met [47].

## 12. DILI versus AIH

Distinguishing DILI from AIH on histology can be an arduous task, since there are no pathognomonic histologic features for either DILI or AIH. None of the proposed scoring systems can differentiate a hepatitic pattern of DILI from acute AIH, as there is significant clinical and histologic overlap [10,35]. Suzuki et al. attempted a histologic distinction between AIH and DILI and proposed that severe Ishak activity scores favored AIH, whereas portal neutrophils and hepatocellular cholestasis were more prevalent in DILI [51]. As described by Dina Tiniakos, patients with DILI often present with centrilobular necrosis with little or no interface hepatitis, which can be a feature of acute AIH as well [10,35]. These patients need to be subjected to a trial with steroid monotherapy. Upon response, the steroids should be quickly tapered off. Patients with DILI will remain in complete remission unless re-exposed to the offending drug. In contrast, patients with AIH will almost always relapse on steroid reduction or withdrawal. It is important to keep such patients under regular follow up for about 5 years, with measurements of liver enzymes and IgG levels, to recognize a possible delayed relapse of AIH [44].

## 13. Management of AIH

Although biopsy may be required for diagnosis, follow up for assessing the stage of disease can be performed through non-invasive tests such as elastography; however, serum-based biomarkers have not been well studied in AIH [51]. Liver biopsy may be repeated after 2 years of biochemical remission and prior to drug withdrawal. Patients without advanced fibrosis or acute fulminant liver failure may be treated using Budesonide or prednisolone and Azathioprine as a second line treatment, and mycophenolate mofetil or tacrolimus can be considered. However, those with cirrhosis or severe/fulminant hepatitis may require liver transplant [52].

## 14. Future Ways Forward and Research

The recent consensus recommendations have proposed criteria for diagnosis of AIH that can be applied to both acute and chronic disease presentations. But there are still several issues that need to be addressed by future prospective studies and trials. Distinction between acute presentation of AIH and DILI is pivotal, since AIH requires long-term immunosuppression, while DILI does not. The histological criteria most useful to differentiate between DILI and AIH in the context of an acute lobular hepatitis remain elusive and need to be evaluated in further studies. In addition, the performance of various activity scoring systems, especially mHAI score, needs to be endorsed in the setting of acute hepatitis, as generally all these scores were designed for evaluation of chronic viral hepatitis. Detailed histopathologic and clinical prospective studies evaluating the specific features distinguishing co-morbidity from severe NASH, as well as from AIH with only NAFLD, as well as the cogency of scoring systems in case of concurrent comorbidities, are required for a better understanding. Validation of these consensus criteria across the different populations and encompassing cases with severe acute and chronic manifestations of AIH are topics for future research.

## Figures and Tables

**Table 1:** Common autoantibodies and their association with autoimmune liver disease.

Antibody	Common Association	Other Associations	Method of Detection
ANA	AIH type1	AIC, PSC, PBC ^#^	IIF
ASMA	AIH type1	AIC, PSC, PBC	IIF, IB, ELISA
LKM-1	AIH type2	HCV	IIF, IB, ELISA
AMA	PBC	Rare- AIH type 1 (low titers)	IIF, IB, ELISA
SLA/LP	AIH	-	IB, ELISA
PANCA	PSC	AIH, PSC	IIF, ELISA
LKM3	AIH type2	-	IIF, IB, ELISA
LC1	AIH type2	HCV	IIF, IB, ELISA

AIH—autoimmune hepatitis, ANA—antinuclear antibodies, ASMA—anti smooth muscle actin, LKM—liver kidney muscle; SLA/LP—soluble liver antigen/liver pancreas, PANCA—perinuclear anti neutrophil cytoplasmic antibodies; LC—liver cytosol, ELISA—enzyme linked immunosorbent assay, IIF—indirect immunofluorescence; IB—immunoblotting, PBC—primary biliary cholangitis, PSC—primary sclerosing cholangitis, AIC—autoimmune cholangitis, HCV—hepatitis C virus; ^#^ In ANA positive cases of PBC–sp100/gp210 may be detected by IB [4].

**Table 2:** Histologic features in the scoring system for diagnosis of autoimmune hepatitis.

1993 Johnson et al. [19]		1999 Alvarez et al. [20]	
Chronic active hepatitis with lobular involvement, piecemeal necrosis, and bridging necrosis	+3	Interface hepatitis	+3
Chronic active hepatitis with piecemeal necrosis without lobular involvement and bridging necrosis	+2	Plasma cells	+1
Rosetting of liver cells	+1	Rosettes	1
Infiltrated with plasma cell predominance	+1	None of the above	−5
Biliary changes	−1	Biliary changes *	−3
Any other changes (e.g., granulomas, siderosis and copper deposits) indicative of a different etiology	−3	Other features	−3

* ‘Biliary changes’ refers to bile duct changes typical of PBC or PSC (i.e., granulomatous cholangitis, or severe concentric periductal fibrosis, with ductopenia) and/or a substantial periportal ductular reaction (so-called marginal bile duct proliferation with a cholangiolitis) with copper/copper-associated protein accumulation.

**Table 3:** Simplified scoring system (SSS) for AIH *.

Parameter	Discriminator	Score
ANA or SMA+	>1:40	+1
ANA or SMA+	>1:80 or	+2
LKM+	>1:40 or	+2
SLA	Positive	+2
IgG level	>Upper limit normal	+1
	>1.1 × upper limit of normal	+2
Liver histology	Compatible	+1
	Typical	+2
Absence of viral hepatitis	No	0
	Yes	+2

* Adapted from Hennes et al. [25]. ≥6 points: probable for AIH >/= 7 points: definite AIH.

**Table 4:** Liver histology for AIH: simplified criteria for the diagnosis of AIH [25].

Typical histology (all three features must be present)	interface hepatitis (lymphocytic/lymphoplasmacytic), emperipolesis, hepatic rosettes	Score 2 points
Compatible Histology	chronic hepatitis with lymphocytic infiltration, no emperipolesis and rosetting	Score 1 point
Atypical Histology	other features—e.g., steatohepatitis	Score 0 points

**Table 5:** Proposed criteria for the histologic scoring of autoimmune hepatitis by Balitzer et al. [28].

Histology score 0	Features not observed in autoimmune hepatitis: florid duct lesion (primary biliary cholangitis), bile duct loss, or copper/CK7 positivity (latter applicable only in cases without any bridging fibrosis)
Histologic score 1 *	(1) Hepatitis with mild or moderate necroinflammatory activity with any one of the following: (a) Ishak A2 (mild/moderate interface activity) (b) Ishak B1 (focal confluent necrosis) (c) Ishak C2 (2–4 foci of lobular activity per 10×) (2) CK7 and negative copper stains (applicable only for cases with Ishak fibrosis score less than 3, this feature is not applicable to acute cases)
Histologic score 2	Hepatitic picture with any one of the following: (1) Numerous plasma cells or in clusters (2) High necroinflammatory activity featuring at least one of the following: (a) Ishak score A3 or higher (at least moderate interface activity) (b) Ishak B2 or higher (confluent necrosis in zone 3 or beyond) (c) Ishak C3 or higher (5 or more foci of lobular activity per × 10)

Adapted from Balitzer et al. [28] * Both (1) and (2) are necessary for a histologic score of 1, except in cases with acute presentation when biliary disease is not a consideration and these stains are not relevant.

**Table 6:** Modified Scoring Criteria proposed by Gurung et al. [33].

Score	Histologic Feature
Typical	Both features of: 1. Prominent plasma cells - Plasma cells account for more than 20% of inflammatory cells), OR - Plasma cell clusters (defined as ≥5 (plasma cells) in lobule or portal tracts 2. Kupffer cell hyaline globules
Compatible	Prominent plasma cells (as defined for ‘typical’ cases) in the absence of Kupffer cell hyaline globules on PAS-D
Atypical	Neither of the two ‘typical’ features or features suggestive of another disease process (e.g., biliary disease or steatohepatitis).

Adapted from Gurung et al. [33].

**Table 7:** Consensus recommendations for histological criteria of AIH from IAIHG, 2020 [35].

Terminology	Portal Hepatitis	Lobular Hepatitis
Likely AIH	Portal lymphoplasmacytic infiltrate PLUS any one of the following features Greater than mild interface hepatitis. Greater than mild lobular inflammation. In the absence of histological suggestion of alternative liver disease	Greater than mild lobular hepatitis (±centrilobular necroinflammation) PLUS at least any one of the following features lymphoplasmacytic infiltrates interface hepatitis portal-based fibrosis In the absence of histological suggestion of alternative liver disease
Possible AIH	Portal lymphoplasmacytic infiltrate Without either of the likely features 1 or 2 above In the absence of histological features suggestive of alternative liver disease OR With one or both of the likely features above In the presence of histological suggestion of alternative liver disease	Any lobular hepatitis (+/−± centrilobular necroinflammation) Without any of the likely features 1–3 above In the absence of histological suggestion of alternative liver disease OR With any of the likely features above In the presence of histological suggestion of alternative liver disease
Unlikely AIH	Portal Hepatitis Without either of the likely features above In the presence of histological suggestion of alternative liver disease	Any lobular hepatitis Without either of the likely features above In the presence of histological suggestion of alternative liver disease

Adapted from Lohse et al. [35].

## Data Availability

Not applicable.

## References

[1] Lv T., Li M., Zeng N., Zhang J., Li S., Chen S., Zhang C., Shan S., Duan W., Wang Q. (2019). Systematic review and meta-analysis on the incidence and prevalence of autoimmune hepatitis in Asian, European, and American population. J. Gastroenterol. Hepatol..

[2] Tunio N.A., Mansoor E., Sheriff M.Z., Cooper G.S., Sclair S.N., Cohen S.M. (2021). Epidemiology of Autoimmune Hepatitis (AIH) in the United States Between 2014 and 2019: A Population-based National Study. J. Clin. Gastroenterol..

[3] Amarapurkar D., Dharod M., Amarapurkar A. (2015). Autoimmune hepatitis in India: Single tertiary referral centre experience. Trop. Gastroenterol..

[4] Sebode M., Weiler-Normann C., Liwinski T., Schramm C. (2018). Autoantibodies in Autoimmune Liver Disease-Clinical and Diagnostic Relevance. Front. Immunol..

[5] Wood I.J., King W.E., Parsons P.J., Perry J.W., Freeman M., Lim-Brick L. (1948). Non-suppurative hepatitis: A study of acute and chronic forms with special reference to biochemical and histological changes. Med. J. Austr..

[6] Mackay I.R. (2008). Historical reflections on autoimmune hepatitis. World J. Gastroenterol..

[7] Kunkel H.G., Ahrens E.H., Eisenmenger W.J., Bongiovanni A.M., Slater R.J. (1951). Extreme hypergammaglobulinemia in young women with liver disease. J. Clin. Investig..

[8] Joske R.A., King W.E. (1955). The L.E.-cell phenomenon in active chronic viral hepatitis. Lancet.

[9] Bearn A.G., Kunkel H.G., Slater R.J. (1956). The problem of chronic liver disease in young women. Am. J. Med..

[10] Tiniakos D.G., Brain J.G., Bury Y.A. (2015). Role of Histopathology in Autoimmune Hepatitis. Dig. Dis..

[11] Washington M.K., Manns M.P., Burt A.D., Portmann B., Ferrel L. (2012). Autoimmune hepatitis. MacSween’s Pathology of the Liver.

[12] Hofer H., Oesterreicher C., Wrba F., Ferenci P., Penner E. (2006). Centrilobular necrosis in autoimmune hepatitis: A histological feature associated with acute clinical presentation. J. Clin. Pathol..

[13] de Boer Y.S., van Nieuwkerk C.M., Witte B.I., Mulder C.J., Bouma G., Bloemena E. (2015). Assessment of the histopathological key features in autoimmune hepatitis. Histopathology.

[14] Miao Q., Bian Z., Tang R., Zhang H., Wang Q., Huang S., Xiao X., Shen L., Qiu D., Krawitt E.L. (2015). Emperipolesis mediated by CD8 T cells is a characteristic histo- pathologic feature of autoimmune hepatitis. Clin. Rev. Allergy Immunol..

[15] Estradas J., Pascual-Ramos V., Martínez B., Uribe M., Torre A. (2009). Autoimmune hepatitis with giant-cell transformation. Ann. Hepatol..

[16] Matta B., Cabello R., Rabinovitz M., Minervini M., Malik S. (2019). Post-infantile giant cell hepatitis: A single center’s experience over 25 years. World J. Hepatol..

[17] Guindi M. (2010). Histology of autoimmune hepatitis and its variants. Clin. Liver Dis..

[18] Ngu J.H., Gearry R.B., Frampton C.M., Stedman C.A. (2013). Predictors of poor outcome in patients with autoimmune hepatitis: A population based study. Hepatology.

[19] Johnson P.J., McFarlane I.G. (1993). Meeting report: International Autoimmune Hepatitis Group. Hepatology.

[20] Alvarez F., Berg P.A., Bianchi F.B., Bianchi L., Burroughs A.K., Cancado E.L., Chapman R.W., Cooksley W.G.E., Czaja A.J., Desmet V.J. (1999). International Autoimmune Hepatitis Group Report: Review of criteria for diagnosis of autoimmune hepatitis. J. Hepatol..

[21] Omagari K., Masuda J., Kato Y., Nakata K., Kanematsu T., Kusumoto Y., Mori I., Furukawa R., Tanioka H., Tajima H. (2000). Re-analysis of clinical features of 89 patients with autoimmune hepatitis using the revised scoring system proposed by the International Auto-immune Hepatitis Group. Intern. Med..

[22] Yatsuji S., Hashimoto E., Kaneda H., Taniai M., Tokushige K., Shiratori K. (2005). Diagnosing autoimmune hepatitis in non-alcoholic fatty liver disease: Is the International Autoimmune Hepatitis Group scoring system useful?. J. Gastroenterol..

[23] Vergani D., Mieli-Vergani G. (2013). Autoimmune manifestations in viral hepatitis. Semin. Immunopathol..

[24] Papamichalis P.A., Zachou K., Koukoulis G.K., Veloni A., Karacosta E.G., Kypri L., Mamaloudis I., Gabeta S., Rigopoulou E.I., Lohse A.W. (2007). The revised international autoimmune hepatitis score in chronic liver diseases including autoimmune hepatitis/overlap syndromes and autoimmune hepatitis with concurrent other liver disorders. J. Autoimmune Dis..

[25] Hennes E.M., Zeniya M., Czaja A.J., Parés A., Dalekos G.N., Krawitt E.L. (2008). International Autoimmune Hepatitis Group. Simplified criteria for the diagnosis of autoimmune hepatitis. Hepatology.

[26] Yeoman A.D., Westbrook R.H., Al-Chalabi T., Carey I., Heaton N.D., Portmann B.C., Heneghan M.A. (2009). Diagnostic value and utility of the simplified International Autoimmune Hepatitis Group (IAIHG) criteria in acute and chronic liver disease. Hepatology.

[27] Czaja A.J. (2011). Comparability of probable and definite autoimmune hepatitis by international diagnostic scoring criteria. Gastroenterology.

[28] Balitzer D., Shafizadeh N., Peters M.G., Ferrell L.D., Alshak N., Kakar S. (2017). Autoimmune hepatitis: Review of histologic features included in the simplified criteria proposed by the international autoimmune hepatitis group and proposal for new histologic criteria. Mod. Pathol..

[29] Fujiwara K., Yasui S., Tawada A., Fukuda Y., Nakano M., Yokosuka O. (2011). Diagnostic value and utility of the simplified International Autoimmune Hepatitis Group criteria in acute-onset autoimmune hepatitis. Liver Int..

[30] Weiler-Normann C., Lohse A.W. (2014). Acute auto- immune hepatitis: Many open questions. J. Hepatol..

[31] Muratori P., Granito A., Lenzi M., Muratori L. (2021). Limitation of the simplified scoring system for the diagnosis of autoimmune Hepatitis with acute onset. Liver Int..

[32] Joshita S., Yoshizawa K., Umemura T., Ohira H., Takahashi A., Harada K., Hiep N.C., Tsuneyama K., Kage M., Nakano M. (2018). Japan Autoimmune Hepatitis Study Group (JAIHSG). Clinical features of autoimmune hepatitis with acute presentation: A Japanese nationwide survey. J. Gastroenterol..

[33] Gurung A., Assis D.N., McCarty T.R., Mitchell K.A., Boyer J.L., Jain D. (2018). Histologic features of autoimmune hepatitis: A critical appraisal. Hum. Pathol..

[34] Tucker S.M., Jonas M.M., Perez-Atayde A.R. (2015). Hyaline droplets in Kupffer cells: A novel diagnostic clue for autoimmune hepatitis. Am. J. Surg. Pathol..

[35] Lohse A.W., Sebode M., Bhathal P.S., Clouston A.D., Dienes H.P., Jain D., Gouw A.S., Guindi M., Kakar S., Kleiner D.E. (2022). Consensus recommendations for histological criteria of autoimmune hepatitis from the International AIH Pathology Group: Results of a workshop on AIH histology hosted by the European Reference Network on Hepatological Diseases and the European Society of Pathology: Results of a workshop on AIH histology hosted by the European Reference Network on Hepatological Diseases and the European Society of Pathology. Liver Int..

[36] Ishak K., Baptista A., Bianchi L., Callea F., De Groote J., Gudat F., Denk H., Desmet V., Korb G., MacSween R.N. (1995). Histological grading and staging of chronic hepatitis. J. Hepatol..

[37] Batts K.P., Ludwig J. (1995). Chronic hepatitis. An update on terminology and reporting. Am. J. Surg. Pathol..

[38] Scheuer P.J. (1991). Classification of chronic viral hepatitis: A need for reassessment. J. Hepatol..

[39] Bedossa P., The French METAVIR cooperative study group (1994). Intraobserver and interobserver variations in liver biopsy interpretation in patients with chronic hepatitis C. Hepatology.

[40] Czaja A.J. (2012). Autoantibody-negative autoimmune hepatitis. Dig. Dis. Sci..

[41] O’Brien C., Joshi S., Feld J.J., Guindi M., Dienes H.P., Heathcote E.J. (2008). Long-term follow-up of antimitochondrial antibody-positive autoimmune hepatitis. Hepatology.

[42] Chazouilleres O., Wendum D., Serfaty L., Montembault S., Rosmorduc O., Poupon R. (1998). Primary biliary cirrhosis–autoimmune hepatitis overlap syndrome: Clinical features and response to therapy. Hepatology.

[43] Tsuneyama K., Baba H., Kikuchi K., Nishida T., Nomoto K., Hayashi S., Miwa S., Nakajima T., Nakanishi Y., Masuda S. (2013). Autoimmune features in metabolic liver disease: A single-center experience and review of the literature. Clin. Rev. Allergy Immunol..

[44] Lohse A.W. (2015). Diagnostic Criteria for Autoimmune Hepatitis: Scores and More. Dig. Dis..

[45] Bach N., Thung S.N., Schaffner F. (1992). The histological features of chronic hepatitis C and autoimmune chronic hepatitis: A comparative analysis. Hepatology.

[46] Muratori P., Granito A., Pappas G., Muratori L. (2009). Validation of simplified diagnostic criteria for autoimmune hepatitis in Italian patients. Hepatology.

[47] De Luca-Johnson J., Wangensteen K.J., Hanson J., Krawitt E., Wilcox R. (2016). Natural history of patients presenting with autoimmune hepatitis and coincident nonalcoholic fatty liver disease. Dig. Dis. Sci..

[48] Ferri P.M., Ferreira A.R., Miranda D.M., e Silva A.C.S. (2012). Diagnostic criteria for autoimmune hepatitis in children: A challenge for pediatric hepatologists. World J. Gastroenterol..

[49] Mileti E., Rosenthal P., Peters M.G. (2012). Validation and modification of simplified diagnostic criteria for autoimmune hepatitis in children. Clin. Gastroenterol. Hepatol..

[50] Arcos-Machancoses J.V., Busoms C.M., Tatis E.J., Bovo M.V., Bernabeu J.Q., Goñi J.J., Martínez V.C., de Carpi J.M. (2019). Development and validation of a new simplified diagnostic scoring system for pediatric autoimmune hepatitis. Dig. Liver Dis..

[51] Suzuki A., Brunt E.M., Kleiner D.E., Miquel R., Smyrk T.C., Andrade R.J., Lucena M.I., Castiella A., Lindor K., Björnsson E. (2011). The use of liver biopsy evaluation in discrimination of idiopathic autoimmune hepatitis versus drug-induced liver injury. Hepatology.

[52] Mack C.L., Adams D., Assis D.N., Kerkar N., Manns M.P., Mayo M.J., Vierling J.M., Alsawas M., Murad M.H., Czaja A.J. (2020). Diagnosis and Management of Autoimmune Hepatitis in Adults and Children: 2019 Practice Guidance and Guidelines from the American Association for the Study of Liver Diseases. Hepatology.

